# Emergency department-initiated palliative care for advanced cancer patients: protocol for a pilot randomized controlled trial

**DOI:** 10.1186/1745-6215-15-251

**Published:** 2014-06-25

**Authors:** Brandon Kandarian, R Sean Morrison, Lynne D Richardson, Joanna Ortiz, Corita R Grudzen

**Affiliations:** 1 Department of Emergency Medicine, Icahn School of Medicine at Mount Sinai, New York, NY, USA; 2 Brookdale Department of Geriatrics and Palliative Medicine, Icahn School of Medicine at Mount Sinai, New York, NY, USA; 3 Department of Emergency Medicine, New York University School of Medicine, Bellevue Hospital, 462 First Avenue, Room A345, New York, NY 10016, USA

**Keywords:** Cancer, Emergency medicine, Palliative care

## Abstract

**Background:**

For patients with advanced cancer, visits to the emergency department (ED) are common. Such patients present to the ED with a specific profile of palliative care needs, including burdensome symptoms such as pain, dyspnea, or vomiting that cannot be controlled in other settings and a lack of well-defined goals of care. The goals of this study are: i) to test the feasibility of recruiting, enrolling, and randomizing patients with serious illness in the ED; and ii) to evaluate the impact of ED-initiated palliative care on health care utilization, quality of life, and survival.

**Methods/Design:**

This is a protocol for a single center parallel, two-arm randomized controlled trial in ED patients with metastatic solid tumors comparing ED-initiated palliative care referral to a control group receiving usual care. We plan to enroll 125 to 150 ED-advanced cancer patients at Mount Sinai Hospital in New York, USA, who meet the following criteria: i) pass a brief cognitive screen; ii) speak fluent English or Spanish; and iii) have never been seen by palliative care. We will use balanced block randomization in groups of 50 to assign patients to the intervention or control group after completion of a baseline questionnaire. All research staff performing assessment or analysis will be blinded to patient assignment. We will measure the impact of the palliative care intervention on the following outcomes: i) timing and rate of palliative care consultation; ii) quality of life and depression at 12 weeks, measured using the *FACT-G* and *PHQ-9*; iii) health care utilization; and iv) length of survival. The primary analysis will be based on intention-to-treat.

**Discussion:**

This pilot randomized controlled trial will test the feasibility of recruiting, enrolling, and randomizing patients with advanced cancer in the ED, and provide a preliminary estimate of the impact of palliative care referral on health care utilization, quality of life, and survival.

**Trial registration:**

Clinical Trials.gov identifier: NCT01358110 (Entered 5/19/2011).

## Background

Palliative care utilizes an interdisciplinary, collaborative, team-based approach to decrease pain and suffering for patients with advanced illness. The goal is to achieve the best possible quality of life, including physical, psychological, social, and spiritual aspects, for patients and families through specific knowledge and skills [1]. Palliative care, as distinct from hospice, is not limited to end-of-life care and is offered simultaneously with life prolonging therapies for persons living with serious chronic illness. It has been shown to significantly improve patient and family member quality of life, while at the same time reducing healthcare costs, improving patient and caregiver satisfaction, reducing distressing symptoms, such as pain or dyspnea, improving quality of care, and reducing hospital length-of-stay and costs per day, thereby reducing overall healthcare expenditures [2-4].

Bringing palliative care into the emergency department (ED), a place designed more to intervene than to comfort, is an important place to begin to make improvements in this area. In addition, offering palliative care services from the ED, at the beginning of the hospital course, might provide even greater benefit to patients, families, and hospitals than inpatient consultation, which often occurs late in a patient’s hospital course [5]. As of 2008, palliative care is an official sub-specialty of Emergency Medicine.

This is a protocol for a single center, parallel, two-arm randomized controlled trial in ED patients with metastatic solid tumors comparing ED-initiated palliative care referral to a control group receiving usual care. We chose not to use a pain management team, social worker, or other supportive care intervention as an attention control as we are interested in whether early comprehensive palliative care consultation impacts quality of life, health care utilization, and survival, not whether this is due to the supportive care aspect of palliative care teams alone. We also made this decision to make the intervention more easily generalizable.

The goals of this study are: i) to test the feasibility of recruiting, enrolling, and randomizing patients with serious illness in the ED and ii) to evaluate the impact of ED-initiated palliative care on health care utilization, quality of life, and survival. To decrease bias in the measurement of baseline and outcome data, all study staff that perform patient follow-up, chart abstraction, or data analyses are blinded to patient assignment. We will measure the impact of the palliative care intervention on the following outcomes: i) timing and rate of palliative care consultation; ii) quality of life and depression at 12 weeks, measured using the *FACT-G* and *PHQ-9*; iii) health care utilization; and iv) length of survival.

## Methods

### Design

To evaluate the impact of ED-initiated palliative care consultation on quality of life, health care utilization, and survival on patients with advanced cancer, we are conducting a patient-level, single center, single blind pilot randomized controlled trial of 125 to 150 participants. The Institutional Review Board at the Icahn School of Medicine at Mount Sinai Hospital approved all study procedures (GCO 08–1234), and every participant provided informed consent.

### Setting

Mount Sinai Hospital is a quaternary care, academic referral center in New York City and its ED is an active, urban emergency department. Annually, approximately 100,000 patient visits are seen in the ED’s Adult and Pediatrics divisions. The ED provides patient care 24 hours per day, seven days per week, to all who seek care. The trial will be conducted in the flow of routine patient care.

### Participants

#### Inclusion criteria

Research coordinators screen the electronic medical record ED track board for patients with our specific advanced cancer staging criteria (Table 1) 8 to 12 hours a day, Monday through Friday. The ED attending on record and the patient’s medical oncologist have to agree before research staff invite the patient to participate. Patients eligible for participation are those with a known advanced cancer that meets our staging criteria who are able to speak English or Spanish fluently, and who are being admitted to or observed in the hospital.

**Table 1:** Cancer staging criteria

**A**	**B**
**Cancer location**	**Cancer type**
Anal	“metastatic”, “mets to”, spread to, recurrent “unresectable”, “locally advanced”, “not a surgical candidate”, Stage IV, other
Brain	Recurrent, relapsed, not a surgical candidate, mets to, refusing surgery/radiation, chemotherapy, other
Breast	“metastatic”, “mets to”, “spread to”, Stage IV, other
Carcinoid	Unresectable, metastatic, mets to, refusing chemo/surgery, not a surgical candidate, Stage IV, other
Cervical	“metastatic”, “mets to”, spread to, recurrent “unresectable”, “not a surgical candidate,” Stage IV, other
Colon/Rectum/Colorectal	“metastatic”, “mets to”, spread to, recurrent “unresectable”, “locally advanced”, “not a surgical candidate”, Stage IV, Dukes D, other
Endometrial/Uterine	“metastatic”, “mets to”, spread to, recurrent “unresectable”, “not a surgical candidate”, Stage IV, other
Esophageal	“metastatic”, “mets to”, spread to, recurrent “unresectable”, “locally advanced”, “not a surgical candidate”, Stage III, Stage IV, other
Gallbladder/Bile duct/Cholangio/Ampullary	“metastatic”, “mets to”, spread to, recurrent “unresectable”, “locally advanced”, “not a surgical candidate”, Stage II, Stage III, Stage IV, other
Kidney/Renal cell	“metastatic”, “mets to”, spread to, recurrent “unresectable”, “not a surgical candidate”, Stage IV, other
Laryngeal/Throat/Nasopharyngeal/Mouth a.k.a Head and Neck	“metastatic to”, “locally advanced”, “spread to regional LN” “refusing surgery/chemo”, “not a surgical candidate”, recurrent, Stage III, Stage IV, other
Liver/Hepatic, Hepatocellular (HCC)	“metastatic”, “mets to”, spread to, recurrent “unresectable”, “locally advanced”, “not a surgical candidate”, “ineligible/not a transplant candidate”, ascites, Stage III, Stage IV, other
Lung or Non-small cell lung cancer (NSCLC)	“unresectable”, “metastatic”, “mets to”, “refusing surgery/chemo” “not a surgical candidate,” Stage IIIb, Stabe IV, recurrent, other
Lung small cell	“metastatic”, “mets to”, “refusing chemo”, recurrent, Extensive Stage, other
Melanoma	“metastatic”, “mets to”, spread to, recurrent, Stage IV, other
Mesothelioma	“unresectable”, “metastatic”, “mets to”, “refusing surgery/chemo,” “not a surgical candidate,” Stage III, Stage IV, recurrent, other
Multiple myeloma	Not a transplant candidate, relapse after transplant, Stage III, Stage IV, other
Osteosarcoma	Unresectable, metastatic, mets to, refusing chemo/surgery, not a surgical candidate, Stage IV, other
Ovarian	“metastatic”, “mets to”, spread to, recurrent “unresectable”, “not a surgical candidate”, Stage III, Stage IV, other
Pancreatic	“metastatic”, “mets to”, spread to, recurrent “unresectable”, “locally advanced”, “not a surgical candidate”, Stage III, Stage IV, other
Penis	Unresectable, metastatic, mets to, refusing chemo/surgery, not a surgical candidate, Stage IV, other
Prostate	“metastatic”, “mets to”, spread to, recurrent, Stage IV, other
Sarcoma	Unresectable, metastatic, mets to, refusing chemo/surgery, not a surgical candidate, not a transplant candidate, relapse after transplant, Stage IV, other
Stomach/Gastric	“metastatic”, “mets to”, spread to, recurrent “unresectable”, “locally advanced”, “not a surgical candidate” (exception for stage II gastric CA, not a surgical candidate is ELIGIBLE), Stage III, Stage IV, other
Thyroid (eligible papillary, follicular, medullary and all anaplastic)	Unresectable, metastatic, mets to, refusing chemo/surgery, not a surgical candidate, Stage IV, other
Vulva	Unresectable, metastatic, mets to, refusing chemo/surgery, not a surgical candidate, Stage IV, other
Other Confirm eligibility with Principal Investigator	“metastatic”, “mets to”, spread to, recurrent “unresectable”, “locally advanced”, “spread to regional lymph node,” “not a surgical candidate”, not a chemo candidate, not a transplant candidate, not a radiation candidate, relapsed, other

#### Exclusion criteria

Patients are excluded if they are unable to answer questions because of severe pain or lethargy, cancer stage is unclear, if they have been seen by palliative care in the past, or if they have evidence of cognitive impairment on the six-item screener [6]. Patients who are planning to leave the immediate geographic area (i.e., move to another state or country) are also excluded.

#### Recruitment

The study was described to ED attendings via email and during grand rounds before patient recruitment began, and staff were informed in real time once recruitment of one of their patients qualified for the study. Oncologists were similarly told about the study before patient recruitment began in person at their faculty meeting, as well as by email. A list of all oncologists with admitting privileges was made, and is continually updated specifying whether the oncologist prefers to be called in advance for every patient. If research staff identify a potential qualifying ED patient based on chart review, s/he discusses the patient with the ED attending and oncologist (if the oncologist requests this). If both agree to allow research staff to approach the patient, the patient is then interviewed at the bedside to ascertain whether they qualify for the study. If the patient meets inclusion criteria, informed consent is obtained in the ED, and patients are offered a $20 gift card as an incentive to participate. Multiple forms of contact (home and mobile numbers, friends and family numbers, and address) are collected to minimize loss of patients to follow-up.

### Interventions

#### Randomization and blinding

**Figure 1:**
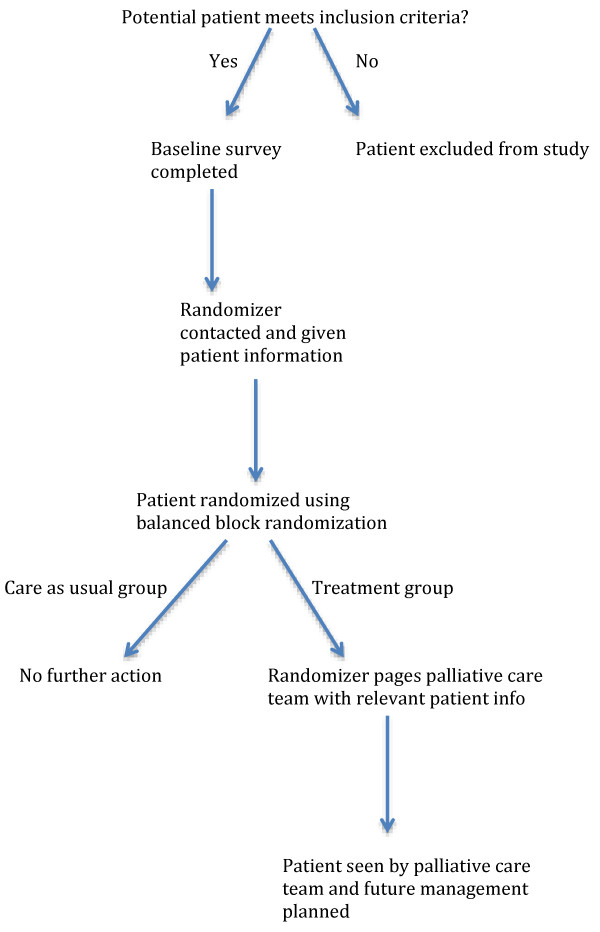
Randomization scheme and participant flow diagram.

After the baseline survey is completed, the research assistant then relays the patient information to a separate research staff member (the “randomizer”) with no role in study recruitment, follow-up, or analysis (see Figure 1 for randomization scheme). Patients are randomized via pre-specified balanced block randomization in blocks of 50. If the patient is assigned to the treatment group, the “randomizer” then pages the palliative care consultation team to relay information about the patient (name, medical record number, ED attending and oncologist of record) and the reason for consultation. If assigned to the care-as-usual group, no further action is necessary. The list linking patient name and group assignment is stored on a secure network computer under password-protection, and accessible only to the “randomizer”. All research staff involved in recruitment and follow-up are blind to patient assignment. It is not feasible to blind patients or care providers to patient assignment.

#### Intervention arm

If the patient is assigned to the intervention arm, the palliative care team is consulted within a few hours. Intervention patients receive a comprehensive palliative care consultation by the inpatient team the same or the following day. At Mount Sinai Hospital, inpatient comprehensive palliative care consultation consists of three components: i) symptom assessment and treatment; ii) establishment of goals of care and advance care plans; and iii) transition planning. The palliative care team is composed of a medical doctor, a nurse practitioner, a social worker, and a chaplain. The team uses validated symptom assessments and makes recommendations for symptom management using National Comprehensive Cancer Network guidelines [7]. They communicate these recommendations to consulting physicians (in this case, the oncologist) using standardized palliative care team chart notes and in person or by telephone. The palliative care team meets with patients, families, and care teams to identify goals of care, complete advance directives, and communicate difficult news (if requested) using standardized communication protocols. If admitted, the team sees patients daily to monitor implementation and results of treatment recommendations and to assess for new and ongoing symptoms. Reassessment and treatment modifications occur as needed to achieve goals of care. The palliative care team conducts or assists with discussions about new or changing goals of care, communicating bad news, and associated treatment adjustments. The team also works with the patients’ social workers and family to facilitate transition management consistent with goals of care. If the team finds ongoing palliative care needs that are expected to continue after discharge, they refer them to the outpatient palliative care clinic.

#### Usual care

Patients assigned to the usual care arm complete the same baseline interviews and follow-up as intervention patients. If requested by the admitting team or oncologist of record, usual care patients may also receive a palliative care consultation.

### Outcome measures

Outcomes were specified ahead of time, and include the rate and timing of palliative care consultation, quality of life and depression at 12 weeks, health care utilization, and survival. Independent variables are collected via a baseline interview with the patient or obtained from the electronic medical record, and are listed in Table 2. Dependent variables are listed in Table 3. The primary outcomes include rate and timing of palliative care consultation, quality of life and depression at 12 weeks, hospital length of stay, and survival. Secondary outcomes include costs during the index admission, ICU admission and ICU days, and ED revisits and re-hospitalization at 30 and 180 days. Objective outcomes were chosen that could be obtained from a chart or administrative data review to eliminate the possibility of recall bias or differential follow-up rates between the intervention and care-as-usual group.

**Table 2:** Independent variables

**Variable**	**Measurement**	**Source**
Treatment group	Intervention, usual care	Research coordinator
Primary MD status	Full-time faculty/hospitalist, voluntary/private practice	Medical Record
Primary MD specialty	Heme/Onc, general medicine, other	Medical Record
Age	Years	Medical Record
Gender	Female, Male	Medical Record
Race/Ethnicity	Asian, Black, Hispanic Black, Hispanic White, Native American, White, Other	Patient Interview
Religion	Catholic, Protestant, Jewish, Muslim, Buddhist, Other, None	Patient Interview
Cancer diagnosis	Tumor type and stage	Medical Record
Performance status	ECOG Performance Status	Patient Interview
Medical comorbidities	Charlson co-morbidity index	Medical Record
Prior living situation	Home, nursing home, hospice	Patient Interview
Use of formal home care	Yes/no, Type of services, h/week	Patient Interview
Primary caregiver	Name and relationship/none	Patient Interview
Advance directives	Living will, health care proxy, power of attorney for health care/none	Patient Interview
Medical insurance	Medicare, Medicaid, Medicaid/Medicare managed care, Medicare managed care, traditional indemnity, self-pay, other	Administrative database, Medical Record

**Table 3:** Dependent variables

**Variable**	**Measurement/Instrument**	**Source**
**Primary Dependent Variables**		
Rate and timing of palliative care consultation	Yes/No; Days from enrollment to consultation	Medical Record
Quality of life	FACT-G at 12 weeks	Patient Interview
Depression	PHQ-9 at 12 weeks	Patient Interview
Hospital length of stay	Days during index admission	Medical Record
Survival	Days from enrollment	Medical Record
**Secondary Dependent Variables**		
ICU Admission and ICU length of stay (if admitted)	Yes/No; ICU bed days	Administrative database
Hospital costs	Total, direct costs during index hospitalization	Administrative database
Hospital readmissions over 30 and 180 days from enrollment	Count	Medical Record
Repeat ED visits over 30 and 180 days from enrollment	Count	Medical Record

### Data collection and management

After the patient is determined to meet inclusion criteria and provides consent, the face-to-face survey is administered in English or Spanish in the ED and takes approximately 15 minutes, depending on responses and interruptions. Research assistants are trained to understand that data collection never interferes with medical care and the interview is stopped for any reason related to their care. The research assistant administers the baseline survey electronically on a table computer using Survey Monkey, an online survey tool. All protected health information is entered into a separate encrypted, password-protected database with only a unique linking identification number to match this information with that entered in Survey Monkey.

The survey includes questions regarding demographics, including gender, race/ethnicity, marital status, income, education, religious affiliation, type of residence, history of an advance directive or designation of a health care proxy, and health insurance; functional status is measured using the Eastern Oncology Cooperative Group score [8]; quality of life measured using the Functional Assessment of Cancer Therapy-General Measure [9]; and the Patient Health Questionnaire-9 [10] is used to screen for depression. Quality of life and depression are measured again at 6 and 12 weeks. The 6-week measurement was added one month into the protocol because a large proportion of our first participants died before the 12-week follow-up.

Six months after the ED visit, outcome data is collected via the electronic medical record and administrative data review using the *Mount Sinai Data Warehouse*. It provides content such as registration data, lab results, medications, radiology reports, procedures, and billing information. For the chart and administrative data review, a codebook was developed, inter-rater reliability was measured, and the research assistant performing chart abstraction was blinded to patient assignment.

### Analysis

We estimated our sample size based on one of our primary outcomes (time to palliative care) by utilizing data from the palliative care database on consultation in patients with advanced cancer. The baseline mean time to consultation for such patients seen by palliative care was 9 days (SD = 12). We estimated that our intervention would decrease this number by at least 50%, to 4.5 days (estimated SD = 6 days). Calculations employ two-tailed tests (α = 0.05, with β = 0.80). We plan to enroll and randomize at least 140 patients. We expect to have at least 80% power with α = 0.05 (two-sided) to detect clinically meaningful differences in these primary outcome variables with 70 subjects/group.

The primary analysis will be a traditional intention-to-treat that will compare intervention to control subjects on our predetermined primary and secondary outcomes. Two sample *t*-tests, Mann–Whitney, or *χ*^2^ will be used as appropriate depending on whether the outcome is continuous or discrete, and whether it is normally distributed. For the quality of life data, where there is a baseline score, ANCOVA will be used to compare outcomes between the two groups, and patients who die will be excluded from this analysis. A series of secondary analyses will be conducted if there is significant contamination between the intervention and control group. In the first of these sets of analyses, randomization assignment will serve as an instrumental variable and percentage of admissions receiving palliative care will be included as a covariate. We will also compare primary and secondary outcomes per protocol (i.e., patients will be grouped according to whether they received a consultation, rather than on their randomization scheme). We expect outliers in our outcome data and will not be excluding them from the analysis. Because of the small sample size, we will not account for permuted blocked randomization in the analyses.

## Discussion

Here, we describe the design of a clinical trial to determine the impact of a palliative care intervention for advanced cancer patients in the ED on quality of life, depression, health care utilization, and survival. While this is a pilot trial to establish feasibility, it will provide an estimate of the effect of the intervention on our predetermined outcomes that can be used to inform the design of a larger multicenter trial.

Systematic reviews of palliative care have concluded that trials are sparse and findings limited due to methodological shortcomings inherent to this patient population [11]. Only a small proportion of the research in palliative care is based on clinical trials [12], as recruitment and retention of these patients for palliative care studies have proven challenging [13]. The ED setting adds other unique challenges not encountered in other settings. The environment is crowded and chaotic, and there is little privacy. Patients often have a high symptom burden and are in significant distress, all of which can preclude participation in research. The specificity of our environment, coupled with the lack of published clinical trials in palliative care, make the publication of our study protocol especially important to further research in this arena.

Visits to the ED for patients with advanced cancer are common, demonstrating the potential for altering the clinical course of these patients in this setting [14,15]. Despite this, there has been almost no needs-based research in palliative care and emergency medicine, and until recently little emphasis has been placed on education, research, or practice guidelines in end-of-life or palliative care in this important setting [16]. While some EDs are pilot testing palliative care programs, there has not been a structured and rigorous approach to the development and testing of such interventions to assure their success and test their impact on predetermined outcomes.

We hypothesize that ED-initiated palliative care consultation will increase the rate and decrease time to palliative care consultation, improve quality of physical and mental health, decrease health care utilization, and may even increase survival. We believe that it is not only feasible to enroll ED patients with serious illness in trials of palliative care, but that the ED presents a unique window in which to do so.

## Trial status

At the time of manuscript submission, the trial is actively enrolling participants.

## Abbreviation

ED: Emergency department.

## Competing interests

The authors declare no competing interests.

## Authors’ contributions

CRG, RSM, and LDR made substantial contributions to conception and design, JMO and BK helped with acquisition of data, and all five authors conducted analysis and interpretation of data. BK drafted the first version of the article and CRG revised it critically for important intellectual content. All authors gave final approval of the version to be submitted and any revised version.
